# Raffinose Inhibits Streptococcus mutans Biofilm Formation by Targeting Glucosyltransferase

**DOI:** 10.1128/spectrum.02076-21

**Published:** 2022-05-16

**Authors:** So-Young Ham, Han-Shin Kim, Eunji Cha, Taehyeung Lim, Youngjoo Byun, Hee-Deung Park

**Affiliations:** a School of Civil, Environmental, and Architectural Engineering, Korea University, Seoul, South Korea; b Division of Biotechnology, College of Environmental and Bioresource Sciences, Jeonbuk National University, Iksan, Jeonbuk, South Korea; c College of Pharmacy, Korea University, Sejong, South Korea; d Biomedical Research Center, Korea University Guro Hospital, Seoul, South Korea; e KU-KIST Graduate School of Converging Science and Technology, Korea University, Seoul, South Korea; Ohio State University

**Keywords:** raffinose, *Streptococcus mutans*, biofilm, glucosyltransferase

## Abstract

Streptococcus mutans is a representative biofilm-forming bacterium that causes dental caries through glucosyltransferase (GTF) activity. Glucans are synthesized from sucrose by GTFs and provide binding sites for S. mutans to adhere tightly to the tooth enamel. Therefore, if a novel compound that interferes with GTF function is developed, biofilm formation control in S. mutans would be possible. We discovered that raffinose, an oligosaccharide from natural products, strongly inhibited biofilm formation, GTF-related gene expression, and glucan production. Furthermore, biofilm inhibition on saliva-coated hydroxyapatite discs through the reduction of bacterial adhesion indicated the applicability of raffinose in oral health. These effects of raffinose appear to be due to its ability to modulate GTF activity in S. mutans. Hence, raffinose may be considered an antibiofilm agent for use as a substance for oral supplies and dental materials to prevent dental caries.

**IMPORTANCE** Dental caries is the most prevalent infectious disease and is expensive to manage. Dental biofilms can be eliminated via mechanical treatment or inhibited using antibiotics. However, bacteria that are not entirely removed or are resistant to antibiotics can still form biofilms. In this study, we found that raffinose inhibited biofilm formation by S. mutans, a causative agent of dental caries, possibly through binding to GtfC. Our findings support the notion that biofilm inhibition by raffinose can be exerted by interference with GTF function, compensating for the shortcomings of existing commercialized antibiofilm methods. Furthermore, raffinose is an ingredient derived from natural products and can be safely utilized in humans; it has no smell and tastes sweet. Therefore, raffinose, which can control S. mutans biofilm formation, has been suggested as a substance for oral supplies and dental materials to prevent dental caries.

## INTRODUCTION

In oral microbial communities, beneficial bacteria are advantageous for oral health, whereas harmful bacteria, which cause various oral diseases, also exist (1). Among oral diseases, dental caries is the most prevalent infectious disease and is expensive to manage (2). Left untreated, cavities can lead to inflammation of the tissue around the teeth, infection or abscess formation, and even loss of teeth (3). Bacteria that cause dental caries are not usually sufficiently concentrated to cause problems. However, when sugar concentrations increase, acidogenic bacteria in oral microbial communities metabolize sugars to organic acids and synthesize polysaccharides (4). Acidic conditions skew the bacterial community toward dominance by aciduric bacteria, and elevated polysaccharide levels promote biochemical and structural changes in the biofilm matrix (dental plaque) (5, 6).

Streptococcus mutans is more viable under acidic conditions than other Streptococcus species, colonizing the human oral cavity environment (7). Streptococcus mutans is a well-known causative bacterium of dental caries and utilizes sugars (sucrose, starch, glucose, and fructose) as sources of carbon (8). Streptococcus mutans secretes glucosyltransferase (GTF) and fructosyltransferase (FTF) (9). These enzymes synthesize glucan and fructan, respectively, using sucrose as a substrate, which damage the tooth enamel due to acid production and formation of biofilms (10).

Streptococcus mutans produces three genetically distinct GTFs (GtfB, GtfC, and GtfD). Each GTF has a unique role but is eventually involved in the formation of dental plaques (11). GtfB and GtfC are associated with initial microbial adherence and the structural stability of the extracellular matrix (12). GtfB synthesizes insoluble glucans rich in α-1,3-glycosidic linkages and is absorbed onto the bacterial cell surface to promote tight cell clustering for microcolony formation. GtfC, which is adsorbed to the enamel within the pellicle, produces a mixture of insoluble and soluble glucans with α-1,6-linkages and serves as an attachment site for bacteria. The role of GtfD in plaque formation remains unclear; however, it synthesizes soluble glucans, which serve as primers for GtfB activity (6, 13, 14).

Overall, GTFs synthesize soluble and insoluble glucans from sucrose, permitting the bacteria to adhere to glucan and subsequently colonize the dental surfaces (12). GTF enzymes are involved in the formation of glucan, a glucose multimer, accounting for 10 to 20% of dental biofilm dry weight (7). Glucan plays an important role in glucan-cell-tooth surface binding by increasing cell aggregation (11). Streptococcus mutans can easily adhere to tooth surfaces and contribute to the formation of the biofilm and its structural integrity (15).

To prevent oral diseases such as dental caries, dental biofilms are often eliminated via nonspecific mechanical removal treatments, such as brushing and flossing, or inhibited by inactivating the growth of cariogenic bacteria through the use of antibiotics (16). Fluoride is the most effective anticaries agent (17); low concentrations of fluoride inactivate a variety of enzymes in intact cells, whereas high concentrations of fluoride enhance the proton permeability of cell membranes as a transmembrane proton carrier. Based on these mechanisms, fluoride affects the production and tolerance of acid and the antimicrobial abilities of S. mutans (18). However, continuous and high concentrations of fluoride treatment cause the emergence of resistant bacteria and fluorosis. Oral bacteria adapted to fluoride treatment exhibit stable resistance to high fluoride levels (19). Furthermore, excess fluoride treatment hypomineralizes tooth enamel by increasing the porosity of the surface and subsurface of tooth enamel (20). This alters the appearance of the tooth, from fine white lines to pitting or staining of enamel. Therefore, other biofilm-inhibiting agents need to be developed to compensate for these drawbacks.

Recently, some researchers have focused on developing biofilm inhibitors that can interfere with the function of GTFs in S. mutans. Many natural compounds (e.g., hops, green tea, traditional medicinal plants, and food extracts) have been used as antibiofilm agents targeting GTFs (21–23). Koo et al. found that propolis collected by Apis mellifera bees is a potent inhibitor of GTF enzymes, with high inhibitory activities against GtfB and GtfC (24). They also discovered that apigenin decreases the accumulation of S. mutans biofilms by affecting the formation of insoluble and soluble glucans in the polysaccharide matrix (25).

We previously reported that raffinose can reduce Pseudomonas aeruginosa biofilm formation by decreasing cellular cyclic diguanylate levels (26). Raffinose is widely present in various vegetables (e.g., ginger, garlic, kohlrabi, onion, parsnip, and scallion) and fruits (e.g., apricot and melon) (27). It is a trisaccharide consisting of galactose, glucose, and fructose. Galactose in raffinose can bind to the LecA protein of P. aeruginosa, which indicates that galactose and raffinose compete for the same binding site of the LecA protein. Similarly, S. mutans contributes to biofilm formation by binding sucrose-derived glucan to GTFs (11). Therefore, the development of novel compounds that interfere with the binding of glucan to GTFs should be considered to inhibit S. mutans biofilm formation.

The objective of this study was to develop a novel S. mutans biofilm inhibitor; we hypothesized that raffinose affects S. mutans biofilm formation by reducing the activity of GTFs. We investigated S. mutans biofilm formation and the molecular mechanisms with raffinose treatment to prove the hypothesis. Biofilm formation was tested under both static and flow conditions, and the antibiofilm mechanism was deduced by estimating sucrose consumption and GtfC binding affinity. GTF-related gene expression levels and glucan production were measured using reverse transcription-quantitative PCR (RT-qPCR) and colorimetric methods, respectively. Furthermore, the applicability of raffinose to dental caries was evaluated using bacterial adhesion tests and scanning electron microscopy (SEM) analysis of biofilms formed on artificial-saliva-coated hydroxyapatite (HA) discs.

## RESULTS

### Effects of raffinose on S. mutans biofilm formation.

**FIG 1:**
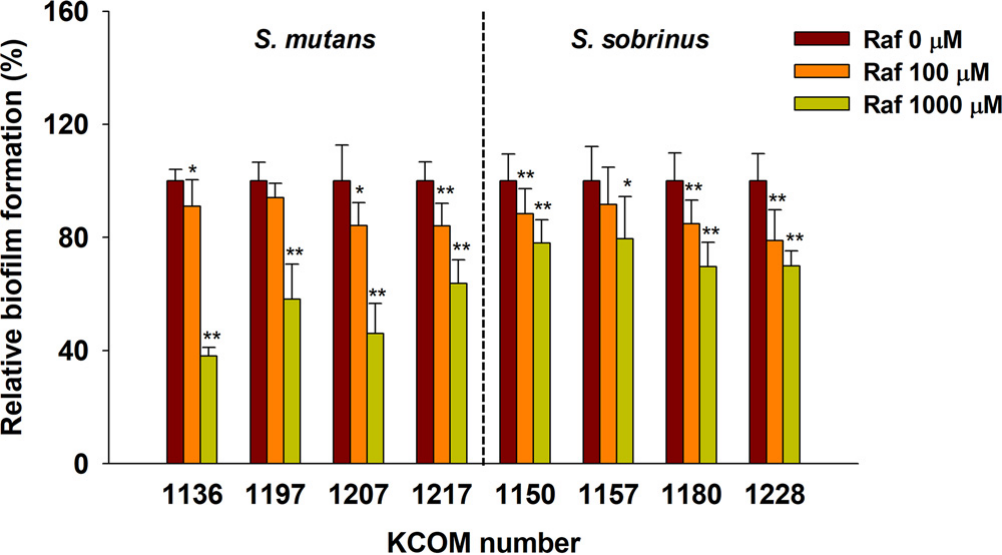
Biofilm formation of Streptococcus species following raffinose treatment for 24 h. Biofilms of S. mutans and S. sobrinus were formed following raffinose treatment (0 to 1,000 μM) under static conditions. Error bars indicate the standard deviations of five measurements. **, *P < *0.005; *, *P < *0.05, versus the control. Raf, raffinose.

Raffinose reduced biofilm formation by most Streptococcus species (KCOM 1136 to KCOM 1228) isolated from human mouths (Fig. 1). Biofilm formation by S. mutans and S. sobrinus was inhibited, on average, by 12 to 25% and 11 to 17%, respectively, compared to untreated biofilm (i.e., control biofilm), when treated with 100 or 1,000 μM raffinose. In particular, biofilm formation by S. mutans KCOM 1136, a representative biofilm-forming bacterium in this study, was decreased most significantly following raffinose treatment, which resulted in biofilm formation being reduced by >50%, compared to control biofilm, after 1,000 μM raffinose treatment.

**FIG 2:**
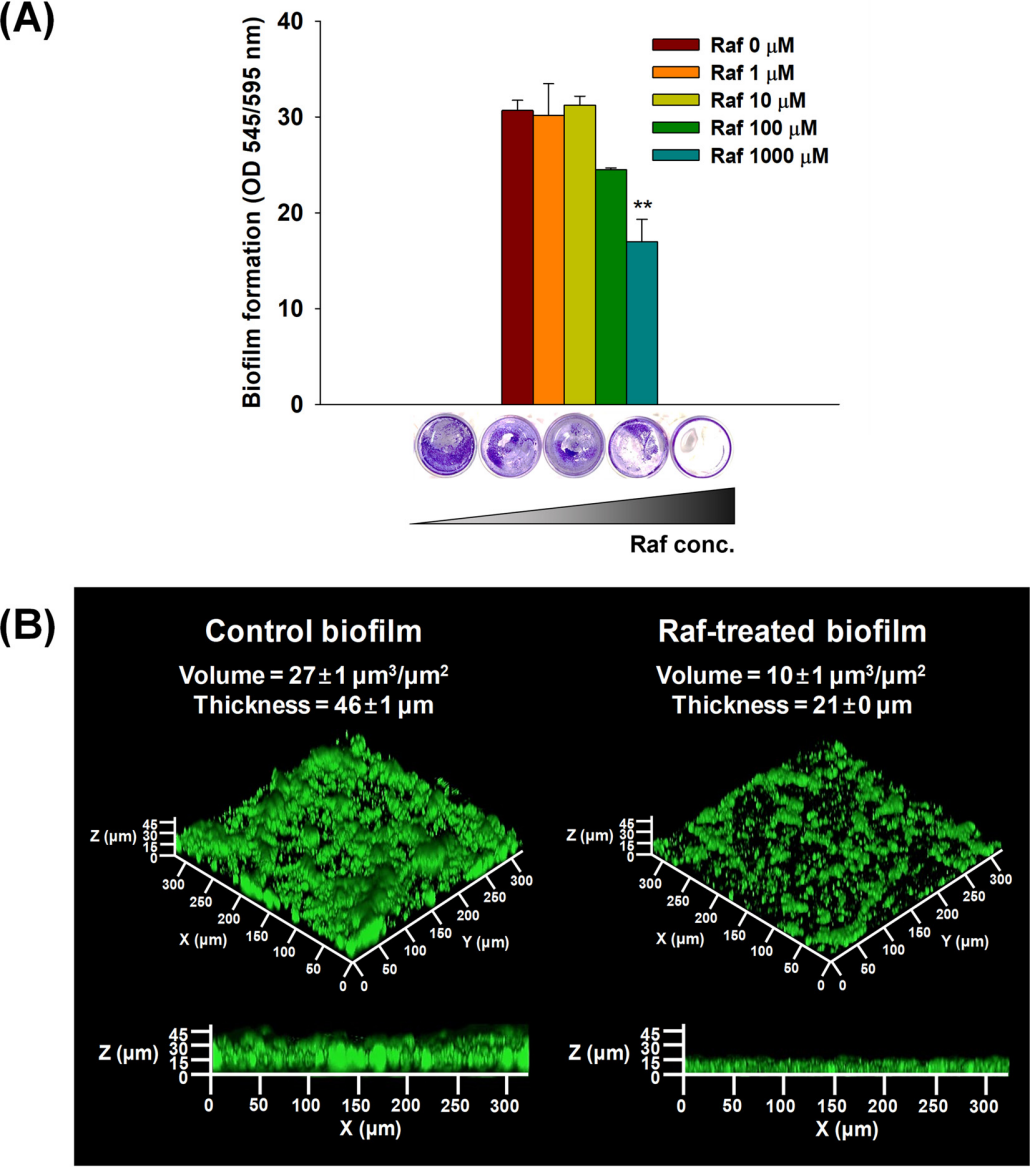
Streptococcus mutans biofilm formation following raffinose treatment under static and flow conditions. (A) CV-stained biofilm following raffinose treatment (0 to 1,000 μM). Quantification was performed by measuring the OD under static conditions (OD_545_/OD_595_). Error bars indicate the standard deviations of five measurements. **, *P < *0.005, versus the control. Raf, raffinose. (B) Volume and thickness of DAPI-stained biofilm based on CLSM images; 1,000 μM raffinose was added to the S. mutans biofilm for 48 h under flow conditions. Raf, Raffinose.

The inhibition of KCOM 1136 biofilm formation by raffinose treatment was analyzed under static and flow conditions. Under static conditions, biofilm formation was dramatically decreased by 44% at a high concentration of raffinose (1,000 μM) (Fig. 2A). Under flow conditions, although the morphology of the biofilm seemed to be similar, with a bumpy shape in control and raffinose-treated biofilms, the average volume and thickness of the raffinose-treated biofilm were decreased by 54 to 64%, compared to the control biofilm (Fig. 2B).

Raffinose is a trisaccharide consisting of galactose and sucrose (see Fig. S1A in the supplemental material). However, contradictory patterns in S. mutans biofilm formation were observed for sucrose and galactose treatments. Galactose exhibited biofilm-inhibiting activity similar to that of raffinose; in contrast, biofilm formation was enhanced in the presence of sucrose (see Fig. S1B). Moreover, sucrose increased S. mutans growth at a concentration of 1,000 μM (see Fig. S2), while there were no differences in the growth of S. mutans with raffinose treatment (0 to 1,000 μM).

### Mechanism of S. mutans biofilm inhibition by raffinose.

**FIG 3:**
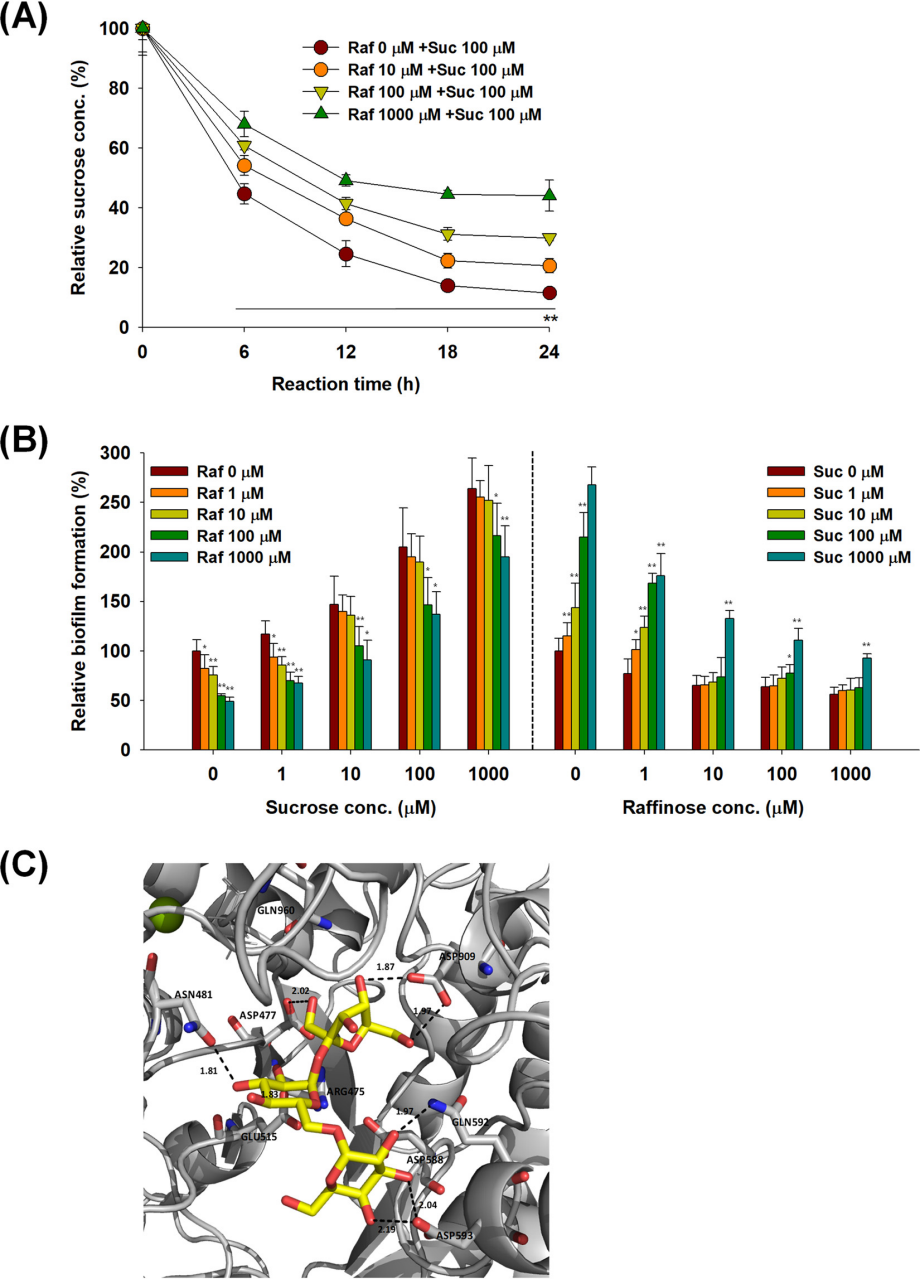
Possible mechanisms underlying the inhibition of Streptococcus mutans biofilm formation following raffinose treatment. (A) Sucrose consumption in S. mutans biofilm cells treated with raffinose. Streptococcus mutans biofilm was formed following sucrose (100 μM) and raffinose (0 to 1,000 μM) treatments under static conditions for 24 h. Error bars indicate the standard deviations of three measurements. **, *P < *0.005, versus the control. Raf, raffinose; Suc, sucrose. (B) Competitive biofilm formation tests between raffinose and sucrose. Streptococcus mutans biofilm was formed following treatment with raffinose and sucrose at concentrations of 0 to 1,000 μM. Error bars indicate the standard deviations of five measurements. **, *P < *0.005; *, *P < *0.05, versus the control. (C) Best-docked poses of raffinose in S. mutans glucansucrase (GtfC [PDB code 3AIC]).

Sucrose consumption of S. mutans following raffinose treatment was monitored for 24 h to investigate the relationship between raffinose and sucrose. The method used for assessment of sucrose consumption was suitable for detecting sucrose concentration but did not respond to high concentrations of raffinose, as indicated in the standard curves for sucrose and raffinose (see Fig. S3). Streptococcus mutans consumed 88% of the sucrose over 24 h (Fig. 3A). However, as the concentrations of raffinose with sucrose in S. mutans increased, sucrose consumption decreased, compared to that in the control (i.e., no raffinose treatment). When 10, 100, and 1,000 μM raffinose was used together with 100 μM sucrose, 79%, 70%, and 56% of the sucrose, respectively, was consumed over 24 h.

Furthermore, competitive biofilm formation was analyzed by adding various concentrations of raffinose and sucrose (0 to 1,000 μM) simultaneously to the culture medium of S. mutans. Biofilm formation decreased by 50% following treatment with 1,000 μM raffinose, whereas it decreased by 26% with simultaneous treatment with 1,000 μM sucrose, compared to that in the control (i.e., no raffinose treatment) (Fig. 3B, left). This finding indicated that the inhibition of biofilm formation following raffinose treatment decreased as the sucrose concentration increased. Similarly, as the raffinose concentration increased, biofilm formation following sucrose treatment decreased (Fig. 3B, right). These results suggest that raffinose and sucrose have a competitive relationship regarding sucrose consumption and biofilm formation in S. mutans.

The possibility of raffinose acting as a glucansucrase inhibitor in S. mutans was investigated using molecular docking studies. Figure 3C shows the best-docked poses of raffinose in the active site of the S. mutans glucansucrase. The docked pose of raffinose was fitted inside the active site of glucansucrase and formed hydrogen bonds with Asp593, a key amino acid residue considered to be the most critical point for acceptor sugar orientation, which influences the transglycosylation specificity of S. mutans glucansucrase (28). However, the docked pose of sucrose was located only in subunit 1, which consists of Arg 475, Asp477, Glu515, and Asp909, without interaction with Asp593, as shown in Fig. S4A in the supplemental material. The glucosyl and fructosyl moieties, which are common in both raffinose and sucrose, made close contact with the amino acid residues of the subunit 1 site. For d-galactose, the best-docked pose was also in close contact only with the amino acid residues of subunit 1, including Asp475, Asp477, Asp909, and Gln 960 (see Fig. S4B). In particular, the OH groups at the C-3 and C-4 positions of the galactose moiety in raffinose interacted with Asp593 as well as with the amino acid residues of the subunit 1 site, which might enhance its binding affinity for glucansucrase. Overall, the docking studies suggested that raffinose, sucrose, and galactose might be in contact with the subunit 1 site of glucansucrase, with total scores of 9.90, 7.88, and 6.42, respectively.

### Effects of raffinose on GTFs in S. mutans.

**FIG 4:**
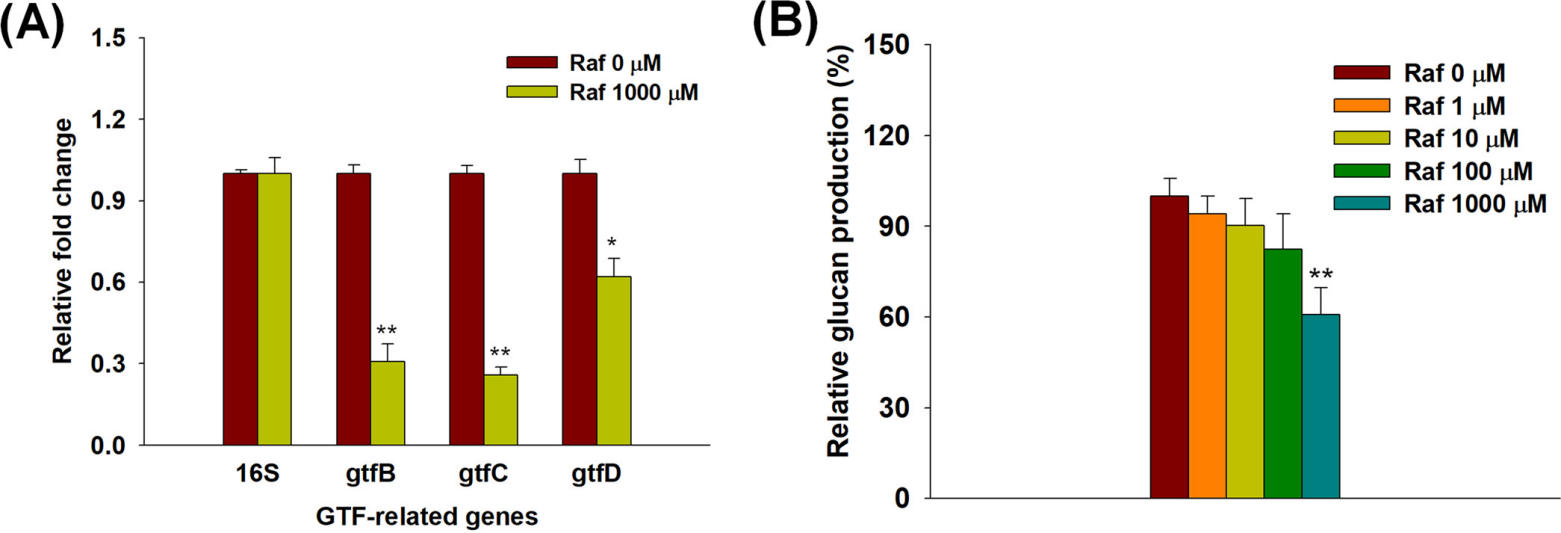
GTF-related changes in Streptococcus mutans following raffinose treatment. (A) GTF gene expression levels in S. mutans biofilm cells. Streptococcus mutans biofilm was formed following raffinose treatment (1,000 μM) under static conditions for 24 h. Relative fold changes were evaluated by RT-qPCR analysis. Error bars indicate the standard deviations of five measurements. **, *P < *0.005; *, *P < *0.05, versus the control. Raf, raffinose. (B) Relative glucan production of S. mutans following raffinose treatment. Extracted insoluble glucan was reacted with raffinose (0 to 1,000 μM). Glucan production was evaluated using the colorimetric method. Error bars indicate the standard deviations of three measurements. **, *P < *0.005, versus the control. Raf, raffinose.

GTF-related gene expression levels and glucan production were analyzed to assess the effects of raffinose on GTFs in S. mutans. As shown in Fig. 4A, all GTF-related genes were significantly downregulated in S. mutans biofilm cells treated with 1,000 μM raffinose. The expression of *gtfB*, *gtfC*, and *gtfD* was repressed by 69%, 74%, and 38%, respectively, compared to that in the control (S. mutans biofilm cells without raffinose treatment). Galactose also evenly decreased GTF-related gene expression by 40 to 43%, whereas sucrose increased the expression levels of GTF-related genes, especially *gtfC* and *gtfD*, by 1.9- to 5.5-fold, compared to those in the control (see Fig. S5A and B). Furthermore, when raffinose and sucrose were used together, most GTF-related gene levels were not significantly different from those in the control (see Fig. S5C). The expression of the 16S rRNA reference gene was not significantly affected by any of the treatments.

**FIG 5:**
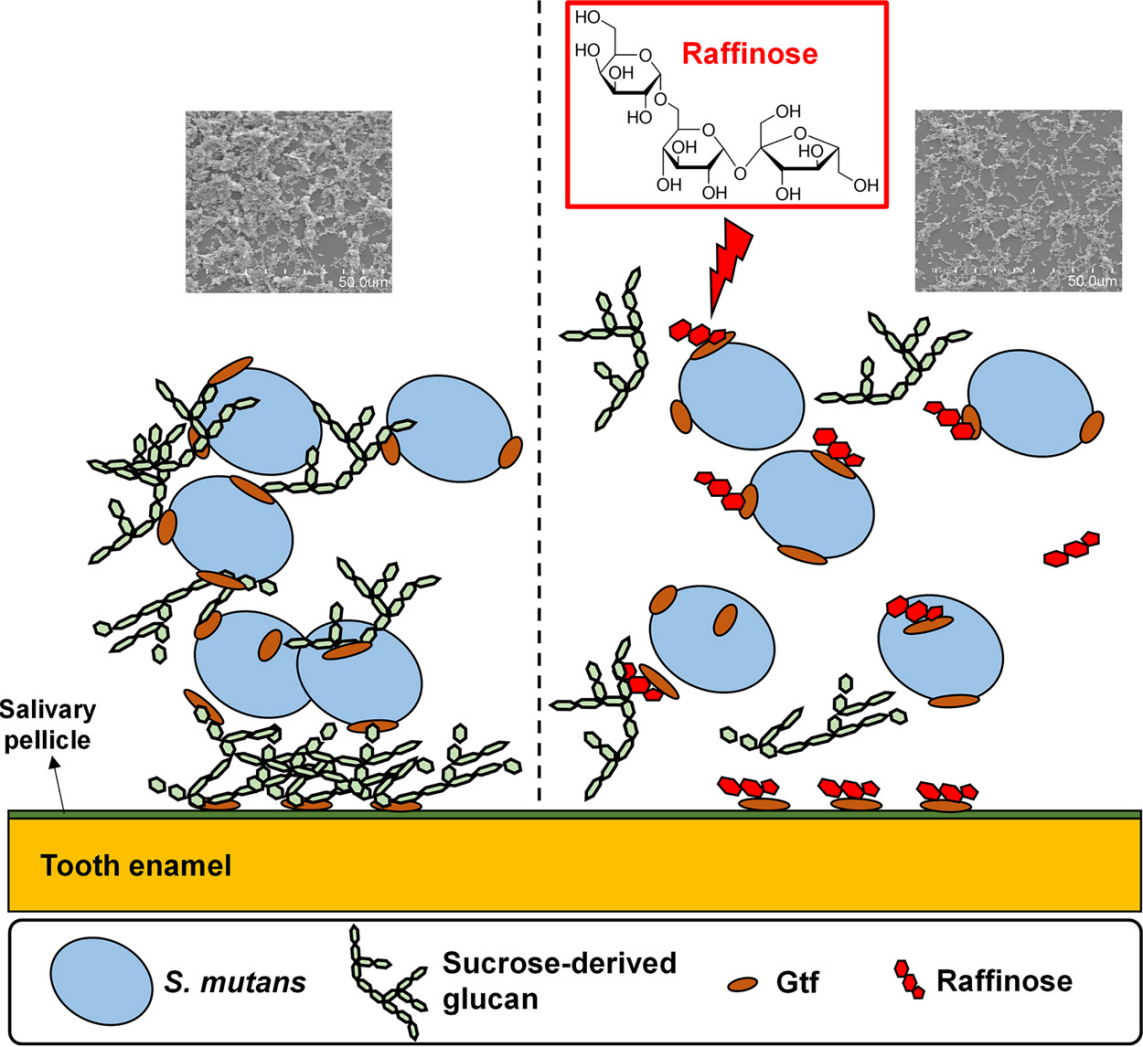
GTF-mediated biofilm inhibition in Streptococcus mutans following raffinose treatment. GTFs secreted by S. mutans are adsorbed on the pellicle and bacterial surfaces. The adsorbed GTFs bind to sucrose-derived glucan. Glucan provides binding sites on the surfaces for S. mutans, mediating adherence to the tooth enamel and tight bacterial clustering and eventually promoting biofilm formation. However, if the S. mutans biofilm is treated with raffinose, then the raffinose is expected to prevent sucrose-derived glucan from binding to GTFs. Therefore, the activity of glucan production in S. mutans is reduced, which may retard biofilm formation.

To evaluate the production of water-insoluble glucans, GTFs were extracted, using solid ammonium sulfate, from S. mutans cultured in brain heart infusion (BHI) medium containing sucrose. The precipitated GTFs were then reacted with raffinose (0 to 1,000 μM). Using sucrose as a substrate, the production of glucans was estimated by measuring the intensity of glucan color using a spectrophotometer. There were no significant changes in glucan production in S. mutans at concentrations below 10 μM raffinose (Fig. 4B). However, glucan production was reduced by 18 to 39% after raffinose treatment at 100 to 1,000 μM, in a concentration-dependent manner. These results implied that raffinose affected the downregulation of GTF-related gene expression and decreased glucan production in S. mutans (Fig. 5).

### Application of raffinose on artificial-saliva-coated HA discs.

**FIG 6:**
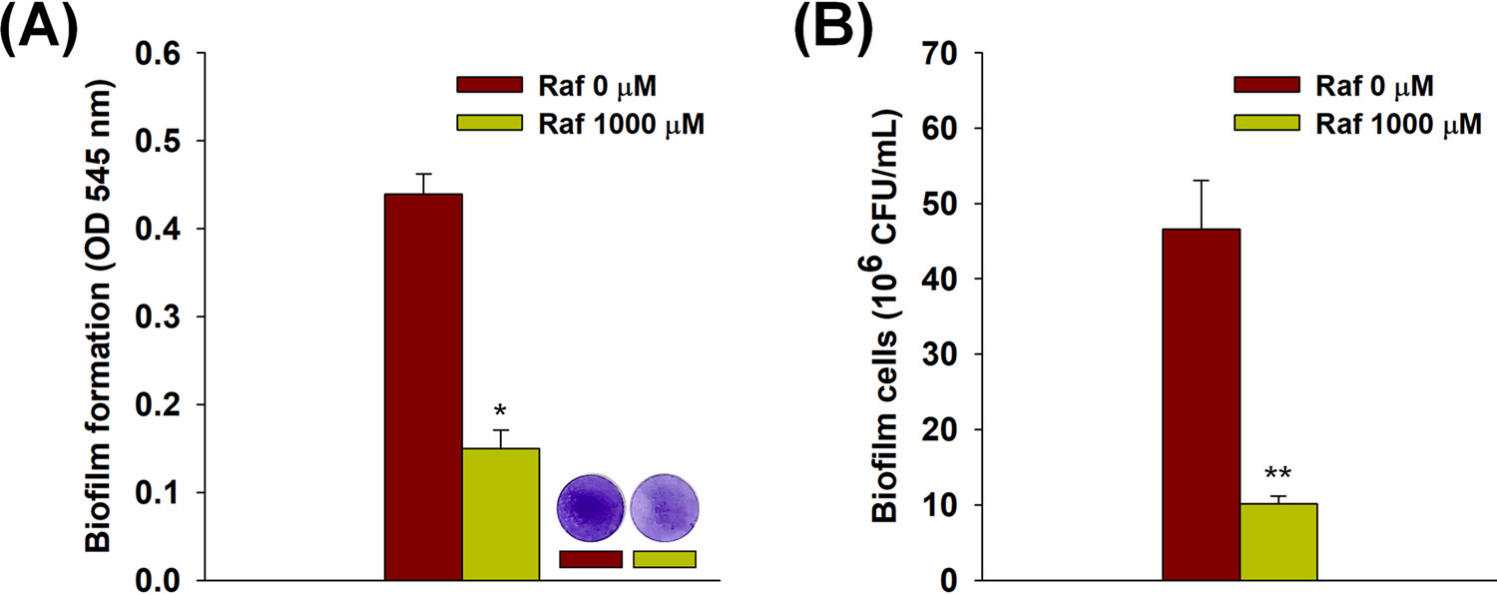
Adhesion of Streptococcus mutans to artificial-saliva-coated HA discs following raffinose treatment (1,000 μM). (A) Bacterial adhesion evaluation using CV staining. Streptococcus mutans biofilm was formed on HA discs for 24 h under static conditions, and OD_545_ was assessed. Error bars indicate the standard deviations of three measurements. *, *P < *0.05, versus the control. Raf, raffinose. (B) Bacterial adhesion evaluation using the cell-counting method. The number of colonies of separated biofilm cells that formed on the HA discs was calculated using the standard plate culture method. Error bars indicate the standard deviations of three measurements. **, *P < *0.005, versus the control.

Artificial-saliva-coated HA discs were used to simulate conditions of the oral environment in humans. Adhesion of S. mutans was evaluated by staining and counting biofilm cells on HA discs. As shown in Fig. 6A, S. mutans biofilm cells on HA discs treated with 1,000 μM raffinose were not stained well, compared to the control, which indicated that S. mutans cells had difficulties in adhering to HA discs after raffinose treatment. Similar results were observed with the colony-counting method, in which the number of colonies on HA discs was diminished following raffinose treatment (Fig. 6B).

**FIG 7:**
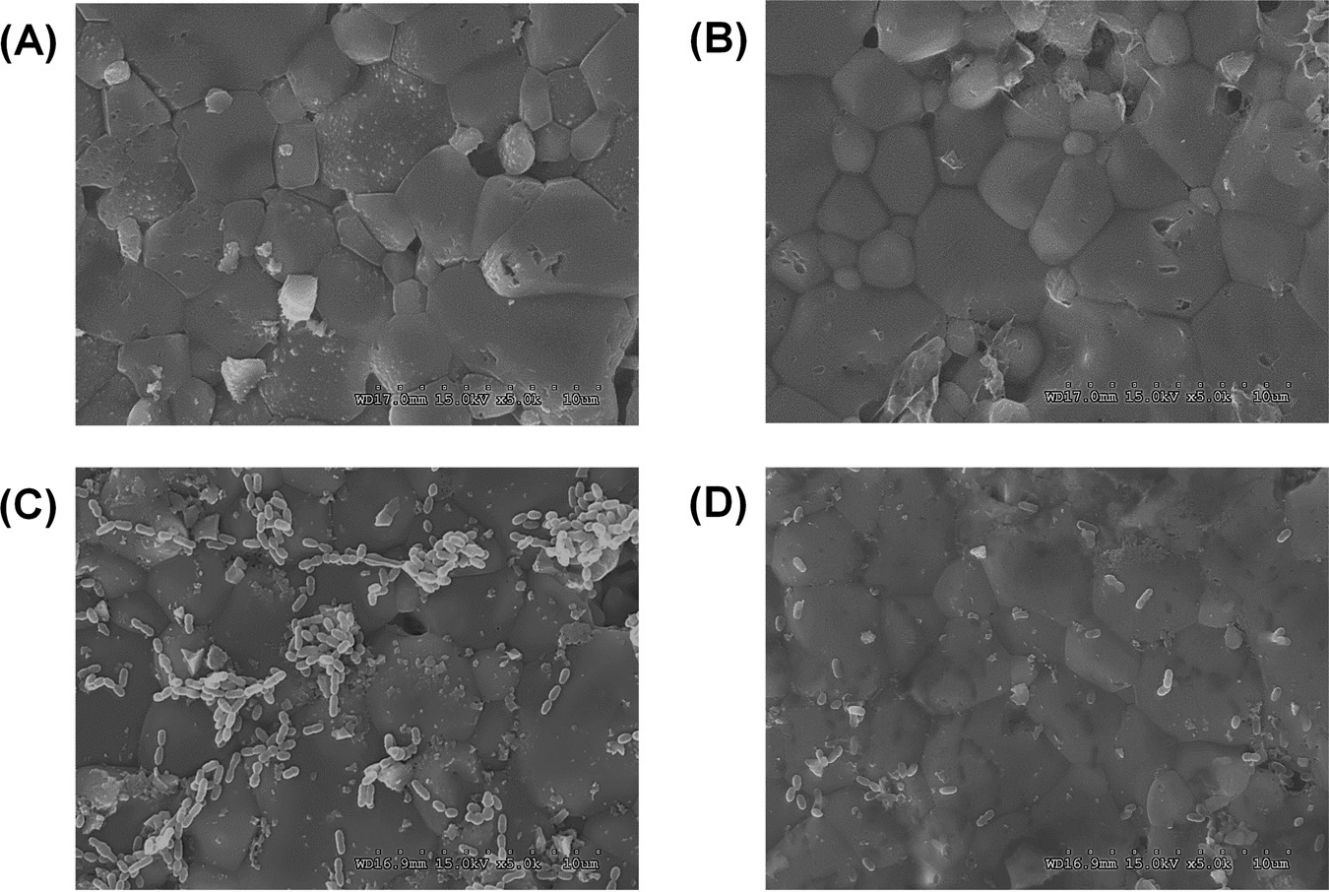
SEM images of Streptococcus mutans biofilm cells following raffinose treatment (1,000 μM) on artificial-saliva-coated HA discs. (A) HA discs. (B) Artificial-saliva-coated HA discs. (C) Streptococcus mutans biofilm cells on artificial-saliva-coated HA discs. (D) Raffinose-treated S. mutans biofilm cells on artificial-saliva-coated HA discs.

The effects of raffinose on S. mutans biofilm formation on HA discs were also demonstrated via SEM analysis. There was no difference between HA discs coated with or without artificial saliva in the SEM images (Fig. 7A and B). Figure 7C and D show SEM images of S. mutans biofilms formed on HA discs. Biofilm formation after 1,000 μM raffinose treatment was reduced significantly, compared to the control (no raffinose treatment). Galactose also adversely affected biofilm formation, whereas sucrose increased it (see Fig. S6). This finding indicates that raffinose can effectively control S. mutans biofilm formation under conditions similar to those of the human oral environment. However, these results do not reliably indicate the applicability of raffinose in the treatment of dental caries. To prevent or treat dental caries with raffinose, further research should be conducted, including clinical demonstration and development of efficient methods for raffinose application.

## DISCUSSION

In this study, we discovered that raffinose reduced biofilm formation by bacteria that cause dental caries in humans. The common cariogenic bacteria, S. mutans and S. sobrinus, adhere to the enamel salivary pellicle and other plaque bacteria to form biofilms (29). Raffinose inhibited S. mutans biofilm formation more effectively than S. sobrinus biofilm formation (Fig. 1). S. mutans is predominantly found in dental biofilm development (74 to 94% of the diverse carious bacterial population), whereas S. sobrinus is less prevalent and is usually detected along with S. mutans (30). S. mutans mainly synthesizes polysaccharides from sucrose as a substrate, contributing to an increase in bacterial cell surface hydrophobicity and biofilm formation (31). Streptococcus mutans biofilm formation increased in response to sucrose, as shown in Fig. S1B in the supplemental material.

Sucrose accelerates S. mutans biofilm formation by targeting GTFs (32). Our RT-qPCR results showed that raffinose mainly downregulated the expression of *gtfB* and *gtfC* in S. mutans (Fig. 4A). However, *gtfD* expression slightly decreased after raffinose treatment. GtfB and GtfC are highly homologous and share 75% amino acid sequence identity in an operon-like arrangement. In contrast, GtfD, which is not linked to the *gtfBC* locus, shares <50% identity with GtfB and GtfC (25). The different patterns for *gtfB-C* and *gtfD* may be related to various factors (carbohydrate availability and source, environmental pH, and growth rate/phase) that influence the transcription of *gtf* genes (25).

Streptococcus mutans synthesizes sticky, water-insoluble/soluble extracellular glucans from sucrose as the sole substrate via the production of GTFs (33). Sticky glucans enhance the pathogenic potential of dental plaque by providing binding sites for S. mutans colonization and promoting accumulation on tooth surfaces. A large proportion of glucans contribute to the establishment of the extracellular polysaccharide matrix and provide bulk and structural integrity to dental biofilms (24, 25). In particular, insoluble glucans are directly related to bacterial attachment during biofilm formation by S. mutans in dental caries. In contrast, soluble glucans indirectly contribute to biofilm formation as a nutrient source for biofilm cells (31). The production of insoluble glucans decreased in proportion to the concentration of raffinose (Fig. 4B).

Most studies regarding biofilm inhibitors for dental caries have focused largely on enzymatic activity in solution, without considering the importance of enzymes adsorbed on the pellicle of the tooth surface. Current commercially available antibiofilm agents show high levels of resistance to surface-adsorbed GTF enzymes; thus, it should be considered that inhibitors of GTF function effectively under conditions similar to those of the oral environment (34). In this study, we used saliva-coated HA, the main constituent of tooth enamel, because GTFs are detected in whole saliva at high levels in individuals with dental caries (11). Furthermore, saliva-coated HA can be tightly bound with GTFs, although GTFs have a low binding affinity for HA and lose their activity over time. As shown in Fig. 7, S. mutans biofilm formation on saliva-coated HA discs was decreased following raffinose treatment.

Although all GTFs interact with saliva-coated HA discs, GtfC has a higher binding affinity, compared with those of GtfB and GtfD (12). GtfC is significantly adsorbed onto the pellicle when exposed to sucrose, thereby enhancing the adherence of bacterial cells to dental surfaces (35). Therefore, inhibition of GTF activities, especially those of GtfC and mainly those adsorbed to the salivary pellicle, is prioritized to prevent the formation of pathogenic dental plaque (24). GtfC secreted by S. mutans is commonly incorporated into the pellicle, and surface-adsorbed GtfC utilizes sucrose to produce glucans (11). Glucans provide binding sites on the surface of S. mutans to easily adhere to tooth enamel, facilitating biofilm formation. In our study, raffinose treatment degraded glucan production, and S. mutans had difficulty adhering to saliva-coated HA discs, eventually resulting in inhibition of biofilm formation on HA discs (Fig. 5).

This phenomenon might be related to the interruption of GTF by raffinose. Streptococcus mutans consumes sucrose to synthesize glucan, thereby increasing biofilm formation (11). When raffinose was combined with sucrose in S. mutans, sucrose consumption and biofilm formation decreased (Fig. 3A), suggesting that raffinose may control GTF activity in S. mutans. In particular, this may be related to the possibility of raffinose binding to GtfC (Fig. 3C). Galactose in raffinose has additional interactions with Asp593, a key amino acid residue, and amino acid residues of the subunit 1 site, which might enhance its binding affinity for glucansucrase. Furthermore, galactose showed inhibitory activities similar to those of raffinose in biofilm formation and GTF gene expression in S. mutans, whereas sucrose showed the opposite effects (Fig. S1B and Fig. S5). Ryu et al. also discovered that galactose significantly inhibits S. mutans biofilm formation by decreasing the expression of three GTF genes (36). These results suggest that galactose may play an important role in the various activities of raffinose in S. mutans. However, these results do not provide direct evidence that raffinose binds to GtfC. Further studies are required to elucidate the detailed mechanism by which raffinose affects GTF activity in S. mutans.

Nagasawa et al. reported that raffinose induces S. mutans biofilm formation with low concentrations of sucrose, which is contrary to our findings (31). Although there may be many reasons for the difference in biofilm formation between the two studies, we cautiously suggest that the difference is correlated with the concentration of raffinose. Streptococcus mutans biofilm formation decreased in proportion to the raffinose concentration (0 to 1,000 μM) in our study. However, the inhibition of S. mutans biofilm formation by raffinose treatment decreased as the sucrose concentration increased (see Fig. S7A). This can be explained by the fact that raffinose is insufficient to inhibit biofilm formation at high levels of sucrose but this does not decrease the activity of raffinose. Conversely, raffinose treatment above 0.03% increases S. mutans biofilm formation (31). A raffinose concentration of 0.03% is equal to 620 μM, which is relatively higher than the raffinose concentrations used in our study. This difference was expected to be due to the remaining raffinose used to inhibit S. mutans biofilm formation. Raffinose can serve as a substrate for FTF in Streptococcus species but is not used by GTF (37–39). Nagasawa et al. also found that raffinose induces S. mutans biofilm formation through fructan synthesis (31). Furthermore, raffinose treatment generally shows biofilm formation patterns; however, at a concentration of less than 0.03% raffinose, there are slight biofilm inhibition patterns. Similarly, in this study, S. mutans biofilm formation increased following treatment with millimolar concentrations of raffinose (see Fig. S7B). Therefore, determination of the raffinose concentrations suitable for environmental conditions should be considered when applying raffinose to regulate S. mutans biofilm formation.

Raffinose, an oligosaccharide found in natural products, greatly reduced S. mutans biofilm formation under static and flow conditions. Furthermore, GTF-related gene expression levels and glucan production were decreased following 1,000 μM raffinose treatment. Moreover, the reduction in bacterial adhesion following raffinose treatment delayed S. mutans biofilm formation on saliva-coated HA discs. The activities of raffinose can be explained by the possibility of binding to GtfC by raffinose. Hence, raffinose has the potential to be utilized as a natural substance to prevent S. mutans biofilm formation in the oral environment.

## MATERIALS AND METHODS

### Bacteria and chemicals.

S. mutans (strain KCOM 1136; Korean Collection for Oral Microbiology [KCOM]) used in this study was clinically isolated and identified in the oral microbiota of Korean subjects. Streptococcus mutans cultured overnight was prepared by incubation with BHI medium (Difco, Detroit, MI, USA) at 37°C in a shaking incubator (200 rpm).

d-(+)-Raffinose pentahydrate, sucrose, and d-(+)-galactose were selected as chemicals that affect S. mutans biofilm formation. These chemicals were purchased from Sigma-Aldrich (St. Louis, MO, USA) and dissolved in dimethyl sulfoxide (DMSO) (Carl Roth, Karlsruhe, Germany).

### Static biofilm formation test.

Streptococcus mutans cultured overnight was adjusted to an optical density at 595 nm (OD_595_) of 1.0 using a UV-visible spectrophotometer (UV mini-1240; Shimadzu, Kyoto, Japan) and then diluted 1:20 (vol/vol) in BHI medium containing 10 μM sucrose. The diluted bacterial culture was grown in the presence of 0 to 1,000 μM sugars for 24 h in 96-well polystyrene microtiter plates (Sigma-Aldrich) and borosilicate bottles. The plates and bottles were incubated at 37°C without agitation, to allow biofilm formation. After 24 h of incubation, the OD_595_ of the suspended cultures in the plates and bottles was measured using an iMark microplate reader (Bio-Rad, Richmond, CA, USA) to analyze the growth of the bacterial cells. The suspended culture was then discarded, and biofilm cells attached to the plates and bottles were washed with phosphate-buffered saline (PBS) (137 mM NaCl, 2.7 mM KCl, 10 mM Na_2_HPO_4_, and 2 mM KH_2_PO_4_ [pH 7.2]) to remove the remaining suspended culture medium. Biofilm cells were stained with 0.1% crystal violet (CV) for 30 min. After the staining period, CV was washed using sterile deionized water to remove the remaining dye and dissolved in 100% ethyl alcohol. The remaining CV was measured at 545 nm using a microplate reader. The extent of CV staining indicated the amount of biofilm formed. Biofilm formation was normalized by dividing OD_545_ by OD_595_.

### Flow biofilm formation test.

To form biofilms under flow conditions, glass slides were inserted into a drip-flow reactor (DFR-110; BioSurface Technologies Corp., Bozeman, MT, USA). BHI medium containing a dilution of S. mutans cultured overnight (OD_595_ of 1.0) with or without raffinose (1,000 μM) was fed into the reactor using a peristaltic pump (Masterflex C/L tubing pump; Cole-Parmer, Vernon Hills, IL, USA) at a flow rate of 0.3 mL/min. The reactor was operated at 37°C for 48 h. At the end of the reactor operation, the slides were carefully removed from the reactor. Biofilms formed on the slides were washed with PBS twice and stained with 4′,6-diamidino-2-phenylindole (DAPI) (Carl Roth) for confocal laser scanning microscopy (CLSM) (LSM 700; Carl Zeiss, Jena, Germany) analysis.

Biofilms were measured in z-stack mode under blue fluorescent light (excitation wavelength, 350 nm; emission wavelength, 470 nm) with a 20× lens objective (W N-Achroplan 20×/0.5 W [DIC] M27). Biofilm morphology was analyzed using ZEN 2011 software (Carl Zeiss), and the volume and thickness of biofilms were measured using the Comstat2 tool of ImageJ software (National Institutes of Health, Bethesda, MD, USA) (40).

### Sucrose consumption test.

A sucrose consumption test was conducted by modifying the method presented in a previous study to suit the experimental conditions (41). A dilution of S. mutans cultured overnight (OD_595_ of 0.05) in BHI medium treated with sucrose (100 μM) and raffinose (10 to 1,000 μM) was aliquoted in borosilicate bottles at 37°C for 24 h. To calculate sucrose consumption, bacterial suspensions were sampled every 6 h and passed through a 0.22-μm membrane filter. Sucrose consumption by the suspension was examined using a sucrose assay kit (Sigma-Aldrich). The filtered suspension (100 μL) was incubated at 25°C for 10 min with 100 μL of sucrose assay reagent. After the addition of 2,000 μL of glucose assay reagent, the reaction mixture was incubated again at 25°C for 15 min. Sucrose consumption was evaluated by measuring the OD_340_ using a spectrophotometer.

### *In silico* docking studies.

All compounds were rendered as two-dimensional (2D) and three-dimensional (3D) structures using ChemDraw Ultra v.12.0.2.1076 and Chem3D Pro v.12.0.2, respectively. Ligand preparation and optimization were performed using the Sanitize preparation protocol in SYBYL-X v.2.1.1 (Tripos Inc., St. Louis, MO, USA). The GtfC protein structure (Protein Data Bank [PDB] code 3AIC) in PDB format was downloaded from the Research Collaboratory for Structural Bioinformatics (RCSB) PDB. The SYBYL-X v.2.1.1 program was employed for protein preparation, including fixation of conflicting side chains of amino acid residues. Water molecules were removed from the protein crystal structure, and chains other than chain A were also removed. Hydrogen atoms were added under the application of AMBER7 FF09 for the force field setting. The minimization process was performed using the POWELL method. The initial optimization option was set to zero. Docking studies involving the ligands were performed using the Surflex-Dock GeomX module in SYBYL-X v.2.1.1. Docking was guided by the Surflex-Dock protocol, and the docking site was defined by the Ligand method with the complexed ligand α-acarbose with a threshold value of 0.50. Other parameters were applied with default settings in all runs.

### RT-qPCR.

A dilution of S. mutans cultured overnight (OD_595_ of 0.05) in BHI medium containing 10 μM sucrose and treated with or without raffinose (1,000 μM) was incubated in borosilicate bottles at 37°C for 24 h. Biofilm samples were collected by scraping bacterial cells attached to the bottles with PBS. Total RNA was extracted from the biofilm samples using TRI Reagent (Molecular Research Center, Cincinnati, OH, USA). RT-qPCR was performed with SYBR Premix Ex Taq (TaKaRa, Shiga, Japan) on a Bio-Rad CFX 96 real-time PCR system (Bio-Rad). The primer sets for GTF-related genes were designed using Primer 3 v.0.4.0 (http://frodo.wi.mit.edu) (see Table S1 in the supplemental material). Thermocycling conditions for the designed primer sets were as follows: initial denaturation at 95°C for 10 s, followed by 40 cycles of denaturation at 95°C for 10 s, annealing at 60°C for 10 s, and extension at 63°C for 34 s. Fluorescent signal intensities were measured at the end of the final extension step. Relative gene expression was normalized to that of 16S rRNA as a reference gene (42) and analyzed using the 2^−ΔΔ^*^CT^* relative expression method (43).

### Glucan production test.

A glucan production test was conducted by modifying the methods used in previous studies (44, 45). A dilution of S. mutans cultured overnight (OD_595_ of 0.01) in BHI medium containing 10 μM sucrose was incubated at 37°C for 24 h with shaking (200 rpm). The supernatant of incubated S. mutans obtained by centrifugation at 4°C for 30 min was precipitated with solid ammonium sulfate (Sigma-Aldrich). After agitation at 4°C for 1 h and centrifugation at 10,000 × *g* at 4°C for 30 min, the precipitate was diluted with 10 mM potassium phosphate buffer (pH 6.0). The extracted crude GTFs were stored at −80°C.

To measure the effects of raffinose treatment on water-insoluble glucan production, mixtures of GTFs treated with raffinose (0 to 1,000 μM) and 20 μL of 0.0625 M potassium phosphate buffer (pH 6.5) were reacted with 12.5 μg/L sucrose and 0.25 μg/L sodium azide (Sigma-Aldrich) and then incubated at 37°C for 24 h. Precipitated water-insoluble glucans were dispersed using a sonicator (VCX 750; SONICS, Newtown, CT, USA) for 4 cycles of 5 s of operation and 5 s of pause, at a frequency of 3.5 Hz. The glucan production was estimated by measuring the OD_550_ using a UV-visible spectrophotometer.

### Bacterial adhesion test.

Dense ceramic HA discs (Clarkson Chromatography Products, Williamsport, PA, USA) were coated with artificial saliva. Following an incubation period at 25°C for 12 h, the HA discs were rotated at 5 rpm for 1 h with artificial saliva. Artificial saliva was prepared according to the ISO/TR10271 standard, i.e., 0.04% sodium chloride (NaCl), 0.08% calcium chloride dihydrate (CaCl_2_·2H_2_O), 0.04% potassium chloride (KCl), 0.0005% sodium sulfide dihydrate (Na_2_S·2H_2_O), 0.08% sodium dihydrogen phosphate (NaH_2_PO_4_·2H_2_O), and 0.1% urea (CH_4_N_2_O) (30). The coated discs were then washed for 30 min with buffered KCl containing 5 mg/mL bovine serum albumin.

A dilution of S. mutans cultured overnight (OD_595_ of 0.01) in BHI medium containing 10 μM sucrose was adhered to artificial-saliva-coated HA discs by incubating the bacterial cells with or without raffinose treatment (0 to 1,000 μM) at 37°C for 1.5 h. Adherent bacterial cells on HA discs were washed twice with PBS and then analyzed using CV staining and cell-counting methods. CV staining was performed as described above for the static biofilm formation test. For cell counting, HA discs were sonicated for 10 min at an amplitude of 30%, using a sonicator, to detach bacterial cells from the discs. The detached bacterial cells were dispersed, diluted, spread on BHI agar plates, and then incubated at 37°C for 24 h. CFU were calculated by counting the number of bacterial colonies formed on agar plates.

### SEM analysis.

A dilution of S. mutans cultured overnight (OD_595_ of 0.05) in BHI medium containing 10 μM sucrose was incubated with glass slides at 37°C for 24 h. Biofilm cells on the slides were fixed with 4% glutaraldehyde solution (Sigma-Aldrich) at 4°C for 1 h. Then, the fixed biofilm cells were sequentially dehydrated in 50%, 80%, and 100% ethyl alcohol for 20 min each. Dehydrated biofilm cells were dried in a vacuum desiccator for 12 h and coated with platinum for 90 s using an E-1030 ion-sputter coater (Hitachi, Tokyo, Japan). The coated sample was observed using a model S-4300 scanning electron microscope (Hitachi) operated at a magnification of ×5,000 and a voltage of 15.0 kV.

### Statistical analysis.

*P* values were estimated via Student's *t* test using SigmaPlot (Systat Software, Inc., San Jose, CA, USA).
