# Supplementary material for: Raffinose Inhibits Streptococcus mutans Biofilm Formation by Targeting Glucosyltransferase
Source: Microbiol Spectr. 2022 May 16;10(3):e02076-21. doi: 10.1128/spectrum.02076-21 (PMC9241737; doi:10.1128/spectrum.02076-21)
Supplement: SUPPLEMENTAL FILE 1 — Supplemental material. Download spectrum.02076-21-s001.pdf, PDF file, 0.7 MB [file spectrum.02076-21-s001.pdf]

**Supplementary Data**

# Raffinose inhibits *Streptococcus mutans* biofilm formation via targeting glucosyltransferase

So-Young Ham[a], Han-Shin Kim[b], Eunji Cha[a], Taehyeung Lim[c], Youngjoo Byun[c,d] and Hee-Deung Park[a,e,*]

[a]School of Civil, Environmental and Architectural Engineering, Korea University, 145 Anam-ro, Seongbuk-Gu, Seoul 02841, Republic of Korea

[b]Division of Biotechnology, College of Environmental and Bioresource Sciences, Jeonbuk National University, Iksan, Jeonbuk, 54596, South Korea

[c]College of Pharmacy, Korea University, Sejong, South Korea

[d]Biomedical Research Center, Korea University Guro Hospital, Seoul, South Korea

[e]KU-KIST Graduate School of Converging Science and Technology, Korea University, Seoul, South Korea

[*]Corresponding Author

- Mail address: School of Civil, Environmental and Architectural Engineering, Korea University, 145 Anam-Ro, Seongbuk-Gu, Seoul 02841, South Korea
- Phone: +82-2-3290-4861
- FAX: +82-2-928-7656
- E-mail: heedeung@korea.ac.kr

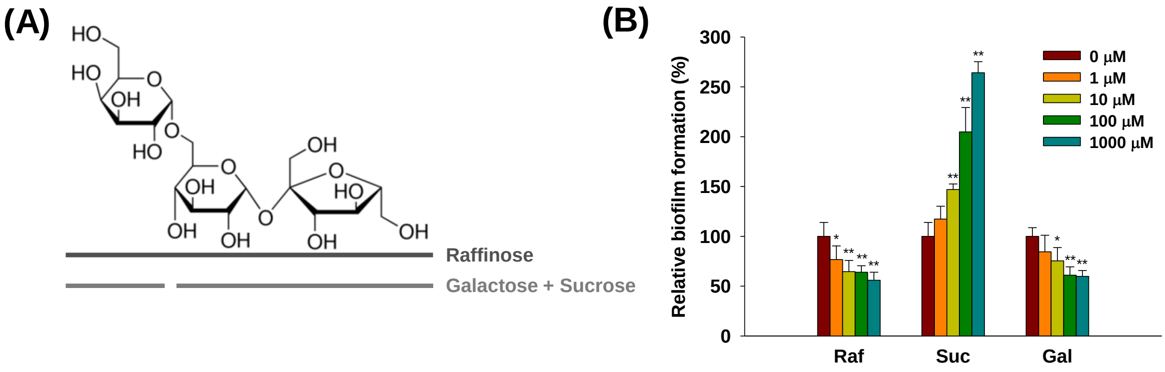


**Figure S1.** Effect of various sugars on *Streptococcus mutans* biofilm formation. (A) Structural comparison of the sugars used in this study. (B) *Streptococcus mutans* biofilm formation following treatment with the indicated sugars. Raffinose, sucrose, and galactose were added at concentrations of 0–1,000 μM. **, $P < 0.005$; *, $P < 0.05$ versus the control. Raf, Raffinose. Suc, Sucrose. Gal, Galactose.

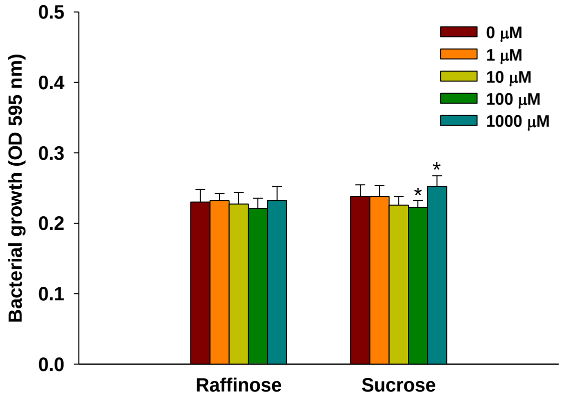


**Figure S2.** The growth of *Streptococcus mutans* with raffinose or sucrose treatment (0–1,000 μM). Error bars indicate the standard deviation of five measurements. **, $P < 0.005$; *, $P < 0.05$ versus the control. OD, optical density.

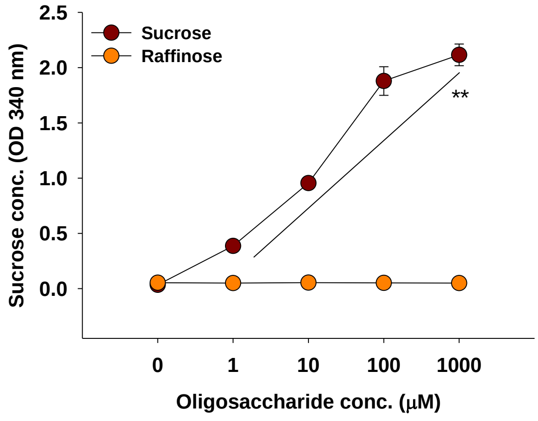


**Figure S3.** Standard curves of sucrose and raffinose (0–1,000 μM) for evaluating the suitability of the sucrose consumption test. Error bars indicate the standard deviation of three measurements. **, $P < 0.005$ versus the control. OD, optical density.

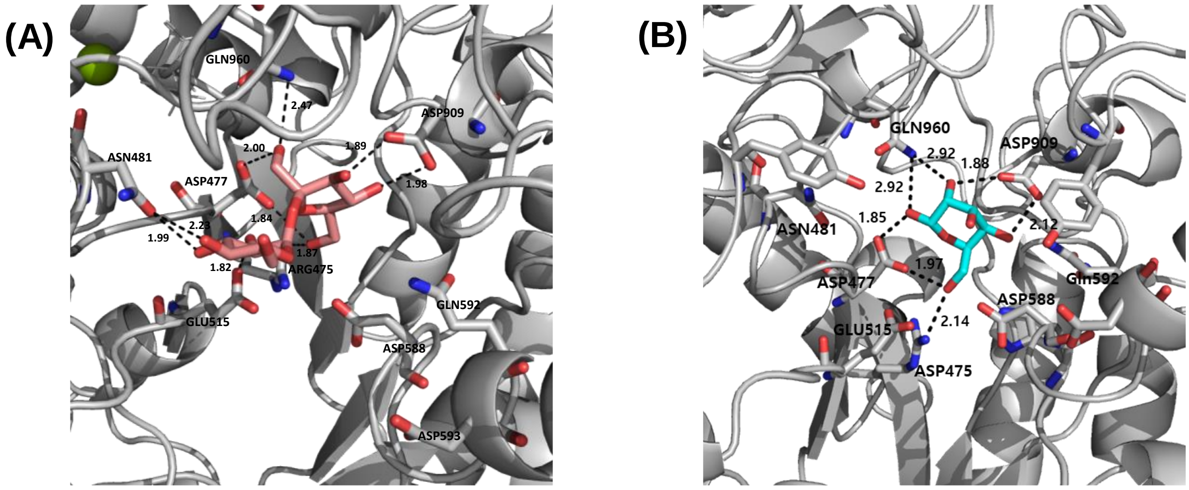


**Figure S4.** The best-docked pose of (A) sucrose and (B) D-galactose in the active site of *Streptococcus mutans* glucansucrase.

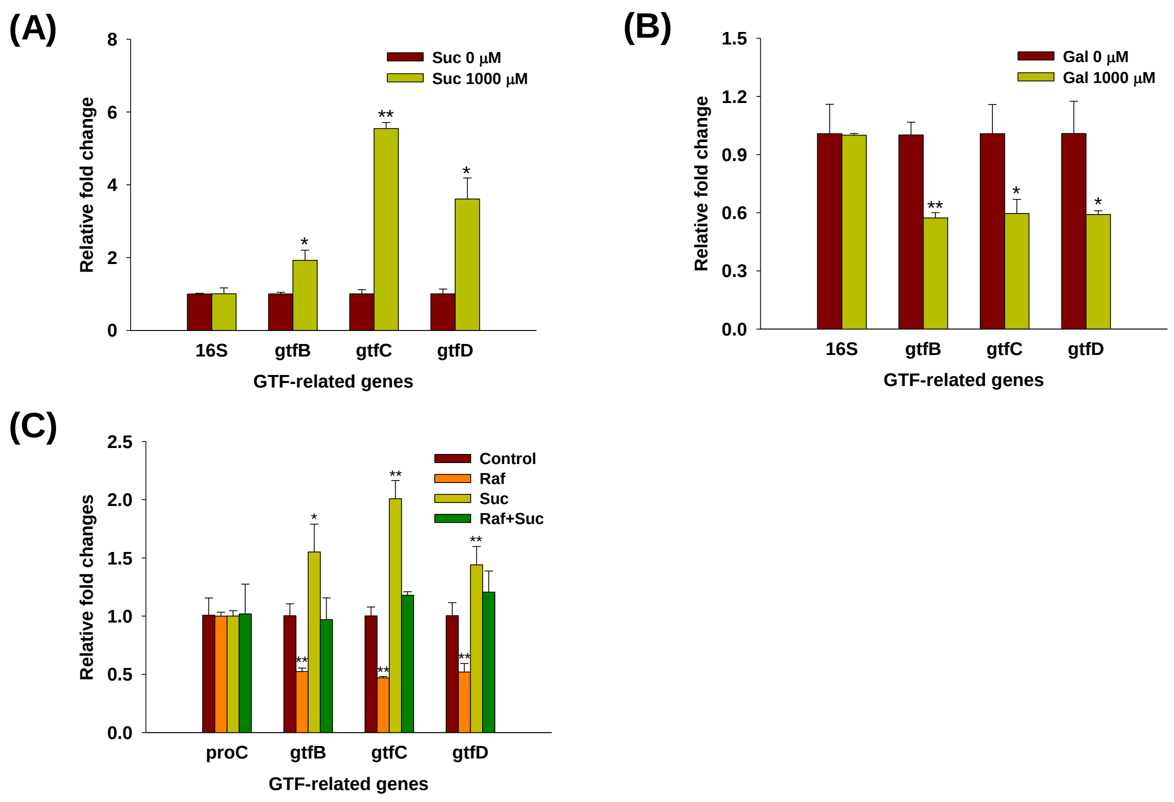


**Figure S5.** Relative glucosyltransferase (GTF)-related gene expression levels in *Streptococcus mutans* biofilm cells following (A) sucrose, (B) galactose, and (C) combined treatment (0–1,000 μM). Error bars indicate the standard deviation of three measurements. **, $P < 0.005$; *, $P < 0.05$ versus the control. Suc, Sucrose. Raf, Raffinose.

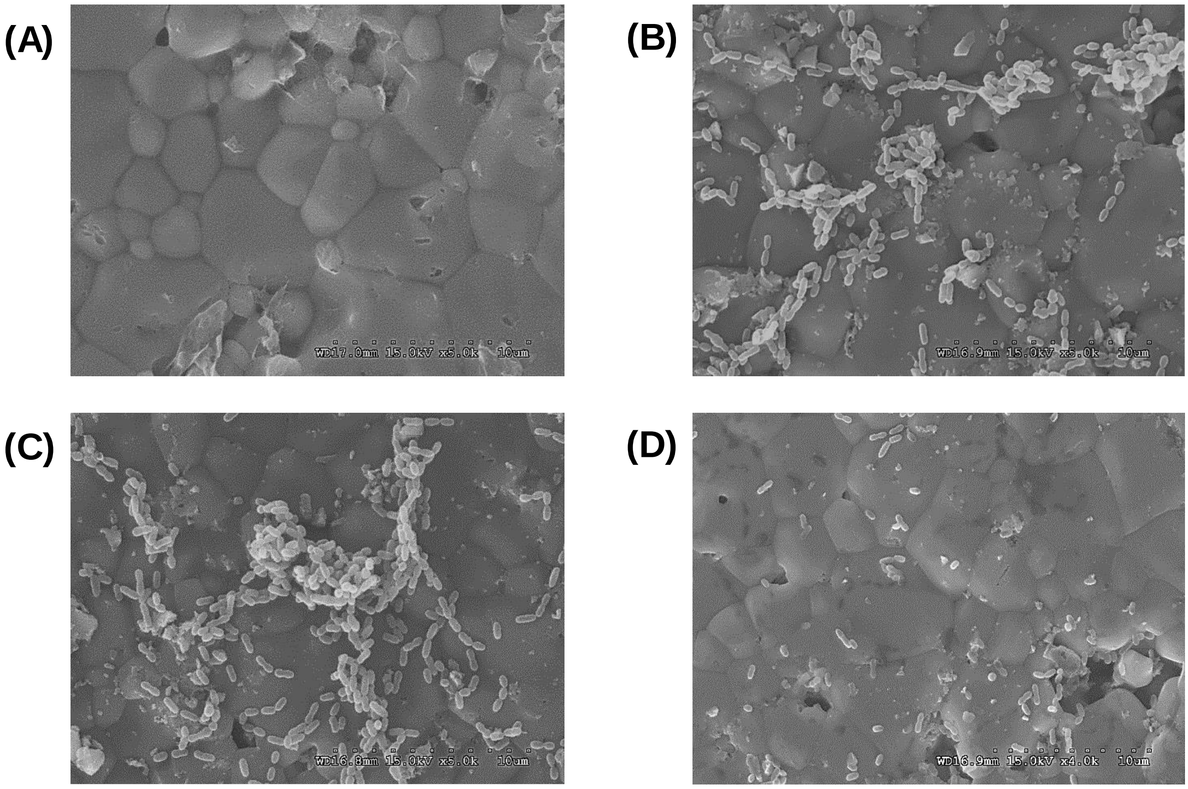


**Figure S6.** Scanning electron microscopy images of *Streptococcus mutans* biofilm after 1,000 μM sucrose and galactose treatment on artificial saliva-coated hydroxyapatite (HA) discs. (A) Artificial saliva-coated HA discs. (B) *Streptococcus mutans* biofilm on artificial saliva-coated HA discs. (C) Sucrose-treated *S. mutans* biofilm on artificial saliva-coated HA discs. (D) Galactose-treated *S. mutans* biofilm on artificial saliva-coated HA discs.

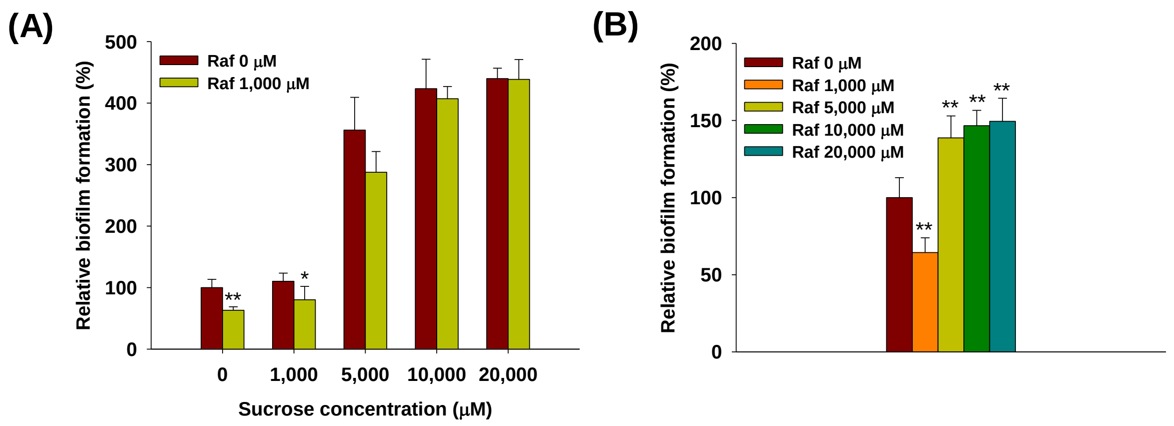


**Figure S7.** Relative *Streptococcus mutans* biofilm formation in the presence of high concentrations (0–20,000 μM) of (A) sucrose and (B) raffinose. Error bars indicate the standard deviations of three measurements. **, $P < 0.005$; *, $P < 0.05$ versus the control. Raf, Raffinose.

**Table S1.** Glucosyltransferase-related primer sets used in this study.

| **Primer name** | **Target gene** | **Sequence (5’ → 3’)** | **GC (%)** | **TM (℃)** | **Reference** |
|---|---|---|---|---|---|
| 16s-F | 16s | CTT ACC AGG TCT TGA CAT CCC G | 54.5 | 62.1 | (1) |
| 16s-R | 16s | ACC CAA CAT CTC ACG ACA CGA G | 54.5 | 62.1 | |
| *gtfB*-F | *gtfB* | GGA CAG CCG ATG ATT TGG | 55.6 | 56.0 | This study |
| *gtfB*-R | *gtfB* | CTT CGC TTG CAG CTC CTC | 61.1 | 58.2 | |
| *gtfC*-F | *gtfC* | CCA TCG AAA CCC TGC TTA AA | 45.0 | 55.3 | This study |
| *gtfC*-R | *gtfC* | GCG ATC AGA AGC CTT CAA AC | 50.0 | 57.3 | |
| *gtfD*-F | *gtfD* | AAC CGC ACA CCA ACC AGT | 55.6 | 56.0 | This study |
| *gtfD*-R | *gtfD* | CAA CGG CAT CAA CAC GAA | 50.0 | 53.7 | |

**References**

1. Rouabhia M, Semlali A. 2021. Electronic cigarette vapor increases *Streptococcus mutans* growth, adhesion, biofilm formation, and expression of the biofilm-associated genes. Oral Dis 27:639-647. https://doi.org/10.1111/odi.13564.
